# Dietary Intake and Physical Activity of Normal Weight and Overweight/Obese Adolescents

**DOI:** 10.1155/2010/785649

**Published:** 2010-05-30

**Authors:** Dina D'Addesa, Laura D'Addezio, Deborah Martone, Laura Censi, Alessandra Scanu, Giulia Cairella, Amedeo Spagnolo, Ettore Menghetti

**Affiliations:** ^1^National Institute for Food and Nutrition Research, Via Ardeatina, 546, 00178 Rome, Italy; ^2^Department of Prevention—Nutrition Unit, Local Health Authority RMB, Via B. Bardanzellu, 8, 00155 Rome, Italy; ^3^Institute of Social Affairs, Via P. S. Mancini, 28, 00196 Rome, Italy; ^4^Study Group of Pediatrics Hypertension, Via P. S. Mancini, 28, 00196 Rome, Italy

## Abstract

*Purpose*. To evaluate the relationship between overweight/obesity and dietary/lifestyle factors among Italian adolescents. *Methods*. On a total of 756 adolescents with mean age 12.4 ± 0.9, body mass index, food consumption, and time dedicated to after school physical activities and to TV viewing were determined. The data were analysed according to age, nutritional status, and gender. The analysis of variance and multiple logistic regression analysis were performed to investigate the association between dietary/lifestyle factors and overweight/obesity. *Results*. The percentages of overweight and obesity were, respectively, 28% and 9% among boys, 24% and 7% among girls. The overweight/obesity condition in both genders was associated with parental overweight/obesity (*P* < .001 for mother), less time devoted to physical activity (*P* < .001 for boys and *P* < .02 for girls) and being on a diet (*P* < .001). Direct associations were also observed between BMI and skipping breakfast and the lower number of meals a day (boys only). *Conclusions*. This pilot study reveals some important dietary and lifestyle behaviour trends among adolescents that assist with identification of specific preventive health actions.

## 1. Introduction

Obesity is considered one of the most remarkable medical and social problem in western societies. In particular, childhood obesity is rapidly emerging as a global epidemic with a great variation in secular trends across countries [1, 2]. It is estimated that in the near future over 26 million children in the European countries will be overweight or obese [3]. This trend will have profound public health consequences since obese children tend to become obese adults [4, 5]. A review of the literature related to the years 1970–1992 revealed that 42–63% of obese children in the USA have become obese adults [6]. Childhood obesity is associated with a plethora of psychosocial disorders [7] and health risks including cardiovascular disease risk factors [8, 9].

In Italy, the National Institute of Research on Food and Nutrition (INRAN) carried out with the collaboration of local public institutions a survey to assess the prevalence of overweight and obesity among 8–10 years old children of 6 regions distributed from North to South during the years 2000–2002. The study demonstrated that 23.9% of children were overweight and 11.1% of the sample were obese [10], highlighting one of the highest prevalence in the European countries [11]. The distribution of obesity was not homogeneous, showing a higher prevalence in the Southern areas compared to that of the Northern areas. Such trends were confirmed by the results of the national survey carried out recently on children of the same age and that showed the following prevalence of overweight and obesity: 23.6% and 12.3% [12]. Paediatric obesity in European countries is a common problem and studies were performed to identify risk factors and action plans for obesity prevention and treatment [11, 13–15]. 

The aetiology of obesity is extremely complex, and it is generally linked to many factors that are not only of genetic type. Most of the time personal lifestyle choices as well as cultural, environmental, and behavioural factors, significantly influence obesity [16, 17]. Research findings have shown that many dietary habits are highly correlated to obesity, and physical activity or other lifestyle behaviours are important covariates [18]. They are considered “universal” risk factors for overweight youth [19] and are identified in the increasingly toxic and obesogenic environments of our societies, specially of developed countries, although their relative causal significance is not well understood [20, 21].

In particular, the eating habits of Italian school aged children and adolescents are involved in a changing process from the more traditional Mediterranean diet, to more westernized eating models, rich in animal proteins, fat, and with low intake of complex carbohydrates and fiber [22–24]. This, together with a decrease in energy expenditure, probably, contributes to the increase of the obesity prevalence [15, 25]. However, most of the studies performed in Italy on school children focused on the estimation of the prevalence of overweight and obesity [11, 12] or on the estimation of the average food and nutrient intakes [22, 23]. The purpose of the present pilot study was to provide data on the association of paediatric overweight and obesity with possible risk factors.

## 2. Materials and Methods

In the years 2004/2006, we studied 756 adolescents (391 males and 365 females), mean age 12.4 ± 0.9, belonging to three public middle schools of a Roman urban area. All students of the selected schools were enrolled on a voluntary basis. Prior to their acceptance, the adolescent's parents or others caregivers were fully informed about the objectives and methods of the study and signed a consent form which included self-reported weight and height and children's weight at birth and if the child had been or not breastfed. The participation rate was 88%. For each student anthropometric, lifestyle and dietary data were collected. Height and weight were measured in triplicate in the school setting, in the morning according to WHO international guidelines [26]. The average of the three measurements was used in the analyses. Weight was measured in underwear by a regularly calibrated battery-operated digital scale (SECA 8129) to the nearest 0.1 kg; height was measured by a portable stadiometer (Promes) to the nearest 0.1 cm. To assess levels of overweight and obesity, we used International Obesity Task Force (IOTF) cut offs [27]. We used BMI because it can be at group level an appropriate index to define overweight in children and adolescents [28]. Information concerning the time dedicated to organized sport activities and to other after school physical activities such as running, jumping, bicycle riding, or playing soccer, and the estimation of the time spent at the computer or watching TV were obtained by means of a questionnaire by an in-depth interview to the students during the anthropometric measurement sessions. The physical activity questions were based on those used for the validated adolescent physical activity recall questionnaire, to investigate on organized sport and nonorganized physical activity out of school and during weekends [29]. The sedentary activity questions we adopted match the ones of the validated adolescent sedentary activity questionnaire [30, 31]. The dietary assessment was based on a 24-hour dietary recall assisted by food records: this method is considered adequate when sample size is sufficiently large [32]. The food questionnaire was delivered to students in the morning at school. The delivery of the “form” was the occasion to gather through recall data on the last meal consumed (breakfast) and for training the adolescents in advance on how to keep complete and accurate records of all food and beverages as they were consumed through the day, without quantification of the portion size. This food registration served solely as a memory prompt during the recall [33] conducted by well trained and standardized dieticians the day after. During the face-to-face interview, the dietician estimated the amount of each food eaten, with the assistance of a visual support that reported standard household measures or portions of the common Italian foods [34]. Another task of the dietician was to collect further details about recipes, food description, preparation practices, and information about students being or not on a diet. The calendar of the food survey was organized in order to represent an adequate proportion of weekdays and weekend days.

Dietary data were carefully checked and entered by interviewers into a nutrition software system INRAN-DIARIO 3.1 developed by INRAN. This system translates the amount of food eaten into individual energy and macronutrients and assigns consumed foods into food groups and subgroups.

## 3. Statistical Analysis

The BMI values were initially recoded into three categories: normoweight, including underweight and normal weight children (the number of underweight children was very small), overweight, and obese. 

Associations between overweight status and physical/sedentary activities (sports activities outside of school, hours of sports activities, hours of playing outside of home, hours of watching TV), lifestyle characteristics (eating breakfast, number of meals per day, being on a diet), energy and macronutrients intakes, and other information (birth weight, breastfeeding, overweight/obesity status of mother and father) were also investigated. Only two categories of BMI values were considered to study these associations, namely, normoweight and overweight, arranging overweight and obese in a single category. The associations between BMI categories and each numerical variable were tested by the Kruskal-Wallis test, while the chi-square test evaluated the associations with categorical variables. All reported probability values (*P* values) were compared to a significant level of  .05. Multiple logistic regression [35, 36] was used to estimate the relationship between students' food habits, lifestyle, and other information, and their likelihood of being overweight by calculating the odds ratios (OR) and the corresponding 95% confidence intervals (CI*s*); *P* values associated with tests for linear trend in the OR were also provided. A first logistic regression model evaluated to what extent the intakes of different food categories were associated with overweight status. For this purpose, tertiles of food intake were calculated for each distribution and the levels of each food item were recoded into *low* consumption if lower or equal to the inferior tertile, *medium* if between the inferior and the superior tertile, and *high* if equal or higher than the superior tertile. OR and associated 95% CI are presented for each level of the variables in comparison with the *high* referent level.

Correlation of each food group with other food groups entered in the model was calculated and the presence of high multicollinearity was excluded. A second logistic regression model evaluated to what extent lifestyle characteristics, energy and macronutrients intakes, birth weight, breastfeeding, being on a diet and parent BMI were associated with overweight status. The number of meals per day was recoded into three categories: *2-3*, *4-5,* and *6-7*. Hours of sports and hours of watching TV were both recoded into two categories (*0–3* and *more than 3, 0–2* and *more than 2, *resp.). Both mother's and father's nutritional status were recoded into *normoweight *(including underweight and normal) and *overweight* (including obesity). Tertiles of distribution were calculated for total daily energy, fats, proteins, and carbohydrates intakes and the levels were recoded into *low*, *medium,* and *high* as described above for food intake.

Variables that showed an association with BMI status in the previous univariate analysis were included in the models, some other independent variables were included through forward selection. All statistical analyses were carried out using the software SAS for windows [37].

## 4. Results

Table 1 presents the distribution of BMI categories by age, stratified by gender. Overall, 28% of the boys and 24% of the girls were overweight; 9% of boys and 7% of girls were obese. Overweight/obesity status showed high rates in 12 and 13 aged boys while it tended to decrease with age in the girls group.

Table 2 summarizes the variables investigated in relation to weight status. Fewer overweight boys played sports outside of school compared with normoweight ones; moreover, overweight students of both genders spent less time in sport activities with respect to the normoweight ones; overweight girls spent more time watching TV; overweight boys ate a little less frequently (4.5 meals a day) than the normoweight ones (about 5 meals a day); fewer overweight boys regularly ate breakfast and more overweight students of both genders were on a diet compared with normoweight subjects. More overweight boys and girls had overweight mother and father compared with the normoweight ones. Overweight boys and girls showed lower intakes of total energy and macronutrients (fat, protein and carbohydrates) than normoweight ones.

Table 3 reports the mean consumption levels of several food categories in relation to children overweight status. Overweight boys reported lower consumption of bread, pasta, salt bakery, milk and yoghurt, sugar-sweetened drinks and sweets, chocolate and jam compared to normoweight ones; overweight girls reported lower consumption of milk and yoghurt compared to normoweight girls.

Results of the logistic regression examining the associations between overweight and food consumption habits are presented in Table 4. The tertiles of intake for each food group entered in the model are reported in Table 5. There was a negative association (*P* < .05) between bread and pasta intake and BMI in boys such that those presenting low and medium level of bread intake were, respectively, 2.76 and 2 times as likely to be overweight than boys presenting high bread intake; similarly, boys presenting low and medium level of pasta intake were, respectively, 1.83 and 2.45 times as likely to be overweight than those presenting high pasta intake. There was a negative relationship between overweight condition and the intake of salt bakery and milk and yoghurt in both genders such that with decreasing consumption there were greater odds of being overweight; similarly, with decreasing intake of meat and meat product (boys only), cheese and eggs (girls only), sugar-sweetened drinks, and sweets chocolate and jams (boys only), there were greater odds of being overweight.

There was a positive association between the overweight status of students and their parents' BMI (Table 6), such that boys with a normoweight mother and father were 0.50 times as likely to be overweight than those with overweight parents; girls with normoweight mother were 0.20 times as likely to be overweight than those with overweight mother, and girls with normoweight father were 0.50 times as likely to be overweight than those having overweight father. There was a negative association between carbohydrates intake and BMI in boys such that those with low intake were 2.5 times as likely to be overweight as those with high intake. No significant associations were found between BMI categories and physical activities, lifestyle characteristics, levels of energy, fat and protein intakes.

## 5. Discussion

The results show a high prevalence of overweight and obesity in the group of adolescents studied. In particular, the mean overweight/obese rates were 37% in boys and 31% in girls with a trend to increase with age in boys (28% at 11 years and 30% at 14–17 years) and to decrease in girls (39% at 11 years and 19% at 14–17 years). The trend of prevalence of overweight/obesity in Roman adolescents reaffirms what other authors have observed in similar population groups [38, 39]. However, the prevalence rates are quite high and underscore the need for public health campaigns aimed at preventing and reducing overweight and obesity in Roman youth.

In this study, overweight status was associated with decreased physical participation (Table 2), highlighting the role that physical activity plays in the childhood obesity epidemic. Time dedicated to sporting activities is significantly higher in normoweight males and females. These results are consistent with the growing body of evidence implicating sedentary activities as one of the leading factors in adolescent overweight [40, 41]. Moreover, our findings showed also associations for hours of television viewing but for girls only, and for sporting activities outside of school for boys only. Strong direct association was also observed between overweight of mother and father and their children, confirming the results of other studies [42, 43]. Regarding birth weight, Hirschler et al. [44] have shown that a high birth weight is linked to a higher risk of becoming obese during childhood, but in our study no relationship was observed between these two variables. As far as breastfeeding is concerned, we did not observe any association with obesity risk reduction; however, since we did not collect information about the duration of breastfeeding, this could be a limit. Nevertheless, on this issue there are contrasting data in the literature [45, 46].

We observed in normoweight subjects a more common habit to have breakfast, but the association is statistically significant only for boys. Our findings could be in accordance with the growing body of evidence supporting the role of this meal in decreasing body weight [47]. Breakfast skipping can lead to overeating later in the day, as it was shown by a study conducted in young healthy men [48]. The result showed the lower frequency of meals consumed associated with the prevalence of overweight status in males, and this agrees with that of other investigators who showed that adolescents with a consistent meal pattern were leaner than those with an inconsistent one [49]. However, this aspect is still controversial [50, 51].

In the present study, the percentage of overweight adolescents who stated to be on a diet is high and it is statistically significant (*P* < .001) when compared with normoweight boys and girls of the same age.

We found a negative relationship between energy, macronutrients intakes, and BMI, so that such consumptions were surprisingly higher in normoweight adolescents, than overweight ones, especially in boys. Different motivations can be addressed to support these results. First, although the normal subjects eat more than overweight students, they have also higher physical activity levels with possible consequent higher energy expenditure [52]. Second, a significant difference resulted in this study between adolescents being on a diet and not being on a diet; 29% and 27%, respectively, of overweight boys and girls reported to be on a diet versus only 3 and 7% of the normoweight ones. Presumably the subjects on a diet had reduced food consumption during the period of the dietary recall. Moreover, as observed in other studies, obese individuals tend to underestimate their food intakes and overestimate their physical activity patterns [53].

Possible underreporting of the studied subjects was preliminary assessed using the criteria defined by Goldberg et al. [54]. The resting metabolic rate (RMR) for each subject was calculated using the prediction equation developed by Commission of the European Communities [55] (RMR[MJ/day] for boys = 0.068 ∗ weight [kg] + 0.57 ∗ height [m] + 2.16; RMR[MJ/day] for girls = 0.035 ∗ weight [kg] + 1.95 ∗ height [m] + 0.84) [56]. The percentage of possible underreporters was almost similar in both normal and overweight. Besides, the mean energy intake was lower in overweight non-underreporting subjects than in normoweight ones. Several food groups' mean intake was lower in overweight/obese than in normoweight non-underreporting subjects showing almost the same results observed in the whole group of subjects. These outcomes suggested not to exclude the possible underreporters from the present analysis.

Changes in dietary patterns in the past few decades, such as an increase in the consumption of high fat and sugar foods, have been also implicated in the increase in obesity [2]. Indeed, in the present study the consumption of food categories or single food items was almost always higher in normoweight boys and girls and it was particularly higher for “milk and yoghurt” (Table 3). In this study, a low “milk and yoghurt” daily consumption was associated with overweight status; this result is on line with several epidemiologic studies where an inverse association has been found between milk and dairy consumption and risk of being overweight [57]. There is an increasing body of literature suggesting that dairy calcium may play a role in maintaining stable body weight [58].

Intake of sugar-sweetened drinks was lower in overweight adolescents. On this matter, evidence implicating a high intake of soft drinks in promoting weight gain is still controversial [59]. Most of our overweight adolescents were on a diet and this could justify the lower consumption of most food items, including sweetened beverages.

The multiple logistic regression analyses of food consumption behavior of boys and girls in relation to overweight status confirmed some of the associations found through the analysis of variance. Boys with a low/moderate consumption of bread, pasta, meat and meat products, sweets, chocolate and jam show a higher tendency towards overweight status with respect to the subjects with high consumption. In both genders, the tendency towards being overweight grows with decreasing intakes of salt bakery and milk and yoghurt. As previously observed, such trends could be explained with the tendency of overweight subjects to be on a diet. 

Parental obesity can be a significant factor predicting the development of obesity in children [42]. Whitaker and colleagues have reported that parental obesity increased the risk of childhood obesity by twofold to threefold at all ages, most likely because the influence of parental obesity is the result of a mixture of genetic and environmental influences.

A positive correlation between adiposity of children and parents was observed, confirming the results obtained by the logistic regression analysis; in particular the tendency towards overweight was 50% lower for students having normoweight father (both genders) and mother (boys only) with respect to those with overweight parents.

## 6. Conclusions

In conclusion, the results of this pilot study on adolescents showed a high percentage of overweight/obesity. Such nutritional status in both genders was associated with parental obesity, low physical activity level, and being on a diet. The possible consequence of being on a diet is the lower consumption in overweight students of most food groups compared to normal weight subjects. Moreover, direct associations were observed between BMI and the more common habit to skip breakfast and a reduced meal frequency (boys only).

From the study emerged some critical features related to the dietary profile and lifestyle of the Roman normal and overweight adolescents studied. It is our intention to deepen and broaden the investigation in order to provide further data useful for identifying specific and preventive public health actions.

##  Competing Interests

The authors declare that they have noncompeting interests.

##  Authors' Contributions

D. D'Addesa conceived, coordinated, participated in the design of the study and drafted the manuscript. L. D'Addezio conceived and performed the statistical analysis and contributed to draft the manuscript. D. Martone participated in the design of the study and to the collection of data. L. Censi conceived and participated in the design of the study and coordinated the collection of anthropometric data. A. Scanu participated to the gathering of nutritional data. G. Cairella participated in the design of the study and to the selection of the studied subjects. A. Spagnolo contributed to perform the statistical analysis. E. Menghetti conceived, coordinated, and participated in the design of the study. All authors read and approved the final version of the manuscript.

## Figures and Tables

**Table 1:** Prevalence of normoweight, overweight and obesity in boys and girls.

Boys		Body Mass Index (kg/m^2^)	Body Mass Index Categories		
Age (years)		Mean ± SD	Normoweight	Overweight	Obese
10-11	(*n* = 97)	19.4 ± 3.1	72%	24%	4%
12	(*n* = 146)	21.6 ± 4.3	54%	32%	14%
13	(*n* = 120)	21.5 ± 3.7	65%	28%	7%
14–17	(*n* = 30)	21.3 ± 3.5	70%	27%	3%

Total	(*n* = 391)	21.0 ± 3.9	63%	28%	9%

Girls		Body Mass Index (kg/m^2^)	Body Mass Index Categories		
Age (years)		Mean ± SD	Normoweight	Overweight	Obese

10-11	(*n* = 104)	20.1 ± 3.5	61%	34%	5%
12	(*n* = 133)	21.1 ± 3.7	68%	23%	9%
13	(*n* = 107)	21.3 ± 3.7	76%	18%	6%
14–17	(*n* = 21)	21.3 ± 2.6	81%	19%	—

Total	(*n* = 365)	20.9 ± 3.7	69%	24%	7%

**Table 2:** Variables investigated in relation to weight status.

	Boys		Girls	
	Normoweight	Overweight	Normoweight	Overweight
Sports activities outside of school, %	80*	68	76	71
Hours of sporting activities, mean ± sd	4.2 ± 3.3**	3.0 ± 2.7	3.0 ± 3*	2.6 ± 2.4
Hours of playing activities outside of home, mean ± sd	5.8 ± 5.4	7.0 ± 6.8	5.0 ± 4.5	6.0 ± 5.7
Hours of watching TV, mean ± sd	2.8 ± 1.4	2.5 ± 1.3	2.4 ± 1.4*	2.7 ± 1.3
Overweight status of mother, %	21**	37	18**	49
Overweight status of father, %	48*	62	50*	68
Birth weight, mean ± sd	3.3 ± 0.6	3.3 ± 0.6	3.2 ± 0.5	3.2 ± 0.5
Breastfeeding, %	73	76	75	73
Eating breakfast, %	89*	77	85	79
Number of meals per day, mean ± sd	4.8 ± 0.7**	4.5 ± 0.8	4.8 ± 0.6	4.7 ± 0.7
Being on a diet, %	3**	29	7**	27
Energy intake, mean ± sd				
(kJ/day)	11608 ± 3888**	10097 ± 3201	9394 ± 2421*	8654 ± 2202
(kcal/day)	2773 ± 929**	2412 ± 765	2244 ± 578*	2067 ± 526
Fat intake (g), mean ± sd	117 ± 46*	105 ± 37	99 ± 32*	89 ± 32
Protein intake (g), mean ± sd	105 ± 39*	94 ± 33	86 ± 27*	80 ± 24
Carbohydrates intake (g), mean ± sd	346 ± 123**	293 ± 104	272 ± 77	253 ± 70

*P < .05 (when compared with the overweight group)

**P < .001 (when compared with the overweight group).

**Table 3:** Consumption of food categories (g/day/per capita, mean ± sd).

	Boys		Girls	
	Normoweight	Overweight	Normoweight	Overweight
Bread	108.7 ± 99*	90.4 ± 83	82.1 ± 69.7	78.4 ± 67.5
Pasta	82.0 ± 62*	64.7 ± 49.3	66.6 ± 59.1	58.6 ± 57.3
Rice	10.6 ± 35.4	14.1 ± 38.2	9.9 ± 31.4	8.4 ± 26.1
Potatoes	58.1 ± 92.9	53.9 ± 98.8	61.4 ± 91.7	54.2 ± 89.7
Salt bakery	70.4 ± 104.7*	55.4 ± 110.9	46.6 ± 75.1	46.8 ± 77.4
Sweet	49.9 ± 55.6	39.8 ± 50.2	41.5 ± 53.2	36.0 ± 42.9
Breakfast cereals	4.7 ± 11.1	3.6 ± 10.5	3.9 ± 10.7	5.8 ± 13.3
Legumes	13.7 ± 36.5	11.5 ± 29.5	11.5 ± 29	9.1 ± 27.3
Vegetables	174.8 ± 124.4	171.3 ± 129.6	148.7 ± 105.9	136.4 ± 115.4
Fresh fruits	131.2 ± 141.5	115.5 ± 138.9	127.8 ± 132	125.5 ± 145.7
Meat and meat products	173.3 ± 123.3	148.0 ± 97.2	149.7 ± 101.4	147.4 ± 99.4
Poultry	47.0 ± 88.6	51.3 ± 90.1	38.3 ± 66.9	51.7 ± 78.8
Fish and seafood	29.4 ± 79.5	36.0 ± 95.2	18.2 ± 47.5	17.8 ± 47.1
Milk and yoghurt	244.9 ± 199.8**	168.9 ± 156.1	197.1 ± 162.4*	155.8 ± 154.7
Cheese	49.2 ± 61.6	56.5 ± 62.9	41.8 ± 53.3	35.5 ± 56.6
Eggs	23.7 ± 39.9	23.7 ± 37.4	22.0 ± 38.9	17.2 ± 34.8
Snacks and potato crisps	4.7 ± 14.8	5.6 ± 22.9	5.5 ± 17.4	4.9 ± 12.7
Sugar-sweetened drinks	301.6 ± 297.8*	237.3 ± 297	183.6 ± 232.4	188.4 ± 234.8
Sweets, chocolate and jam	28.2 ± 33.9**	14.7 ± 20.2	20.0 ± 25.2	19.0 ± 23.5

*P < .05 (when compared with the overweight group)

**P < .001 (when compared with the overweight group).

**Table 4:** Food consumption in relation to overweight status: results of multiple logistic regression.

		Odds ratio	95% confidence limits		*P*
Boys	Bread (low versus high)	**2.76**	1.50	5.01	**.001**
	Bread (medium versus high)	**2.00**	1.11	3.57	**.02**
	Pasta (low versus high)	**1.83**	1.03	3.26	**.03**
	Pasta (medium versus high)	**2.45**	1.38	4.38	**.002**
	Salt bakery (low versus high)	1.55	0.76	3.18	.226
	Salt bakery (medium versus high)	**2.38**	1.37	4.11	**.002**
	Breakfast cereals (low versus high)	0.67	0.33	1.35	.266
	Meat and meat products (medium versus high)	**2.15**	1.22	3.78	**.008**
	Milk and yoghurt (low versus high)	**2.36**	1.28	4.35	**.005**
	Cheese (low versus high)	0.65	0.36	1.18	.157
	Eggs (low versus high)	0.70	0.36	1.36	.295
	Sugar-sweetened drinks (low versus high)	2.20	1.25	3.86	**.006**
	Sweets, chocolate and jam (low versus high)	**2.81**	1.58	5.01	<**.001**
	Sweets, chocolate and jam (medium versus high)	**2.05**	1.13	3.72	**.01**

Girls	Bread (low versus high)	0.97	0.52	1.81	.929
	Bread (medium versus high)	1.65	0.89	3.07	.111
	Pasta (low versus high)	1.46	0.81	2.64	.208
	Pasta (medium versus high)	0.70	0.37	1.30	1.325
	Salt bakery (low versus high)	**1.87**	1.02	3.43	**.04**
	Salt bakery (medium versus high)	1.41	0.71	2.78	.325
	Breakfast cereals (low versus high)	0.37	0.19	0.76	.067
	Meat and meat products (medium versus high)	1.07	0.58	1.95	.833
	Milk and yoghurt (low versus high)	**2.42**	1.24	4.71	**.009**
	Cheese (low versus high)	**2.28**	1.22	4.26	**.009**
	Eggs (low versus high)	**1.98**	1.08	3.63	**.03**
	Sugar-sweetened drinks (low versus high)	0.64	0.34	1.19	.159
	Sweets, chocolate and jam (low versus high)	1.16	0.63	2.13	.641
	Sweets, chocolate and jam (medium versus high)	0.72	0.39	1.30	.270

**Table 5:** Nutrients and food categories' tertiles of consumption (g/day/per capita).

	Inferior tertile	Superior tertile
Energy intake (kJ)	8639.5	10915.2
Fat intake (g)	85.9	114.4
Protein intake (g)	77.7	101.4
Carbohydrates intake (g)	250.5	320.9
Bread	45	112.8
Pasta	46.8	89.2
Salt bakery	0	50
Breakfast cereals	0	1.2
Meat and meat products	102.4	187.3
Milk and yoghurt	124.5	251.6
Cheese	6.8	54.6
Eggs	0	14.7
Sugar-sweetened drinks	7.8	250
Sweets, chocolate and jam	0	23

**Table 6:** Lifestyle characteristics and other information in relation to overweight status: results of multiple logistic regression.

		Odds ratio	95% confidence interval		*P*
Boys	Sports activities outside of school (*yes* versus *no*)	0.73	0.36	1.45	.371
	Hours of sporting activities (*0–3* versus *more than three*)	1.35	0.74	2.46	.325
	Hours of watching TV (*0–2* versus *more than two*)	1.31	0.82	2.10	.255
	Overweight status of mother (*normoweight* versus *overweight*)	**0.55**	0.33	0.94	**.029**
	Overweight status of father (*normoweight* versus *overweight*)	**0.57**	0.36	0.93	**.024**
	Eating breakfast (*yes* versus *no*)	0.65	0.32	1.32	.232
	Number of meals per day (*2-3* versus *6-7*)	2.57	0.76	8.68	.127
	Number of meals per day (*4-5* versus *6-7*)	1.64	0.77	3.47	.20
	Energy intake (kJ) (*low* versus *high*)	0.89	0.25	3.22	.864
	Fat intake (g) (*low* versus *high*)	1.16	0.43	3.12	.764
	Protein intake (g) (*low* versus *high*)	1.22	0.49	3.09	.668
	Carbohydrates intake (g) (*low* versus *high*)	**2.53**	1.09	5.86	**.029**

Girls	Sports activities outside of school (*yes* versus *no*)	1.02	0.54	1.94	.105
	Hours of sporting activities (*0–3* versus *more than three*)	1.66	0.90	3.08	.071
	Hours of watching TV (*0–2* versus *more than two*)	0.79	0.47	1.35	.394
	Overweight status of mother (*normoweight* versus *overweight*)	**0.24**	0.14	0.41	<**.0001**
	Overweight status of father (*normoweight* versus *overweight*)	**0.50**	0.30	0.85	**.009**
	Eating breakfast (*yes* versus *no*)	0.94	0.47	1.89	.859
	Number of meals per day (*2-3* versus *6-7*)	1.13	0.24	5.30	.876
	Number of meals per day (*4-5* versus *6-7*)	0.76	0.33	1.73	.512
	Energy intake (kJ) (*low* versus *high*)	0.96	0.19	4.83	.962
	Fat intake (g) (*low* versus *high*)	1.27	0.38	4.26	.698
	Protein intake (g) (*low* versus *high*)	1.04	0.42	2.59	.933
	Carbohydrates intake (g) (*low* versus *high*)	2.19	0.72	6.67	.167

## References

[1] Wang Y, Lobstein T (2006). Worldwide trends in childhood overweight and obesity.. *International Journal of Pediatric Obesity*.

[2] (2003). WHO: diet, nutrition and the prevention of chronic diseases.

[3] Jackson-Leach R, Lobstein T (2006). Estimated burden of paediatric obesity and co-morbidities in Europe—part 1. The increase in the prevalence of child obesity in Europe is itself increasing. *International Journal of Pediatric Obesity*.

[4] Whitaker RC, Wright JA, Pepe MS, Seidel KD, Dietz WH (1997). Predicting obesity in young adulthood from childhood and parental obesity. *New England Journal of Medicine*.

[5] Deshmukh-Taskar P, Nicklas TA, Morales M, Yang S-J, Zakeri I, Berenson GS (2006). Tracking of overweight status from childhood to young adulthood: the Bogalusa Heart Study. *European Journal of Clinical Nutrition*.

[6] Serdula MK, Ivery D, Coates RJ, Freedman DS, Williamson DF, Byers T (1993). Do obese children become obese adults? A review of the literature. *Preventive Medicine*.

[7] Pierce JW, Wardle J (1997). Cause and effect beliefs and self-esteem of overweight children. *Journal of Child Psychology and Psychiatry*.

[8] Baker JL, Olsen LW, Sørensen TI (2007). Childhood body-mass index and the risk of coronary heart disease in adulthood. *New England Journal of Medicine*.

[9] Bjørge T, Engeland A, Tverdal A, Smith GD (2008). Body mass index in adolescence in relation to cause-specific mortality: a follow-up of 230,000 Norwegian adolescents. *American Journal of Epidemiology*.

[10] INRAN (2003). L’esperienza italiana: progetto pilota basato sui dati locali. *Manuale di Sorveglianza Nutrizionale*.

[11] Lobstein T, Baur L, Uauy R (2004). Obesity in children and young people: a crisis in public health. *Obesity Reviews*.

[12] Spinelli A, Lamberti A, Baglio G, Andreozzi S, Galeone eD Okkio alla salute: sistema di sorveglianza su alimentazione e attività fisica nei bambini della scuola primaria. http://www.okkioallasalute.it/.

[13] Papandreou D, Malindretos P, Rousso I (2010). Risk factors for childhood obesity in a Greek paediatric population. *Public Health Nutrition*.

[14] Reinehr T, Widhalm K, L’Allemand D, Wiegand S, Wabitsch M, Holl RW (2009). Two-year follow-up in 21,784 overweight children and adolescents with lifestyle intervention. *Obesity*.

[15] Roman B, Serra-Majem L, Pérez-Rodrigo C, Drobnic F, Segura R (2009). Physical activity in children and youth in Spain: future actions for obesity prevention. *Nutrition Reviews*.

[16] Sinha A, Kling S (2009). A review of adolescent obesity: prevalence, etiology, and treatment. *Obesity Surgery*.

[17] Boyington JE, Carter-Edwards L, Piehl M, Hutson J, Langdon D, McManus S (2008). Cultural attitudes toward weight, diet, and physical activity among overweight African American girls. *Preventing Chronic Disease*.

[18] Jebb SA (2007). Dietary determinants of obesity. *Obesity Reviews*.

[19] Guo SS, Chumlea WC (1999). Tracking of body mass index in children in relation to overweight in adulthood. *American Journal of Clinical Nutrition*.

[20] Brownell KD, Fairburn CG, Brownell KD (2002). The environmental and obesity. *Eating Disorders and Obesity*.

[21] Elinder LS, Jansson M (2009). Obesogenic environments—aspects on measurement and indicators. *Public Health Nutrition*.

[22] Bellù R, Riva E, Ortisi MT (1996). Preliminary results of a nutritional survey in a sample of 35 000 Italian schoolchildren. *Journal of International Medical Research*.

[23] Leclercq C, Piccinelli R, Arcella D, Le Donne C (2004). Food consumption and nutrient intake in a sample of Italian secondary school students: results from the INRAN-RM-2001 food survey. *International Journal of Food Sciences and Nutrition*.

[24] Verduci E, Radaelli G, Stival G, Salvioni M, Giovannini M, Scaglioni S (2007). Dietary macronutrient intake during the first 10 years of life in a cohort of Italian children. *Journal of Pediatric Gastroenterology and Nutrition*.

[25] Panagiotakos DB, Chrysohoou C, Pitsavos C, Stefanadis C (2006). Association between the prevalence of obesity and adherence to the Mediterranean diet: the ATTICA study. *Nutrition*.

[26] WHO (1995). Physical status: the use and interpretation of anthropometry. Report of a WHO Expert Committee.

[27] Cole TJ, Bellizzi MC, Flegal KM, Dietz WH (2000). Establishing a standard definition for child overweight and obesity worldwide: international survey. *British Medical Journal*.

[28] Bellizzi MC, Dietz WH (1999). Workshop on childhood obesity: summary of the discussion. *American Journal of Clinical Nutrition*.

[29] Booth ML, Okely AD, Chey T, Bauman A (2002). The reliability and validity of the adolescent physical activity recall questionnaire. *Medicine and Science in Sports and Exercise*.

[30] Hardy LL, Booth ML, Okely AD (2007). The reliability of the Adolescent Sedentary Activity Questionnaire (ASAQ). *Preventive Medicine*.

[31] McHale SM, Crouter AC, Tucker CJ (2001). Free-time activities in middle childhood: links with adjustment in early adolescence. *Child Development*.

[32] Buzzard M, Willett W (1998). 24-hour dietary recall and food record methods. *Nutritional Epidemiology*.

[33] Lytle LA, Nichaman MZ, Obarzanek E (1993). Validation of 24-hour recalls assisted by food records in third-grade children. The CATCH Collaborative Group. *Journal of the American Dietetic Association*.

[34] Istituto Scotti Bassani (1990). *Atlante Ragionato di Alimentazione*.

[35] Scott M (2002). Applied logistic regression analysis. *Quantitative Applications in the Social Sciences*.

[36] Agresti A (1996). *An Introduction to Categorical Data Analysis*.

[37] SAS Institute Inc (1985). *SAS User’s Guide: Statistics, Version 5*.

[38] Bellizzi M, Horgan G, Guillaume M, Dietz W, Chen C, Dietz W (2001). Prevalence of childhood and adolescent overweight and obesity in Asian and European countries. *Obesity in Childhood and Adolescence*.

[39] Janssen I, Katzmarzyk PT, Boyce WF, King MA, Pickett W (2004). Overweight and obesity in Canadian adolescents and their associations with dietary habits and physical activity patterns. *Journal of Adolescent Health*.

[40] Storey ML, Forshee RA, Weaver AR, Sansalone WR (2003). Demographic and lifestyle factors associated with body mass index among children and adolescents. *International Journal of Food Sciences and Nutrition*.

[41] Forshee RA, Anderson PA, Storey ML (2004). The role of beverage consumption, physical activity, sedentary behavior, and demographics on body mass index of adolescents. *International Journal of Food Sciences and Nutrition*.

[42] Strauss RS, Knight J (1999). Influence of the home environment on the development of obesity in children. *Pediatrics*.

[43] Bukara-Radujković G, Zdravković D (2008). Determinants of body mass index in children and adolescents. *Srpski Arhiv za Celokupno Lekarstvo*.

[44] Hirschler V, Bugna J, Roque M, Gilligan T, Gonzalez C (2008). Does low birth weight predict obesity/overweight and metabolic syndrome in elementary school children?. *Archives of Medical Research*.

[45] Cope MB, Allison DB (2008). Critical review of the World Health Organization’s (WHO) 2007 report on “evidence of the long-term effects of breastfeeding: systematic reviews and meta-analysis” with respect to obesity. *Obesity Reviews*.

[46] Gillman MW, Rifas-Shiman SL, Berkey CS (2006). Breast-feeding and overweight in adolescence: within-family analysis. *Epidemiology*.

[47] Rampersaud GC, Pereira MA, Girard BL, Adams J, Metzl JD (2005). Breakfast habits, nutritional status, body weight, and academic performance in children and adolescents. *Journal of the American Dietetic Association*.

[48] Martin A, Normand S, Sothier M, Peyrat J, Louche-Pelissier C, Laville M (2000). Is advice for breakfast consumption justified? Results from a short-term dietary and metabolic experiment in young healthy men. *British Journal of Nutrition*.

[49] Ma Y, Bertone ER, Stanek EJ (2003). Association between eating patterns and obesity in a free-living US adult population. *American Journal of Epidemiology*.

[50] Rolland-Cachera MF, Bellisle F (2002). *Child and Adolescent Obesity. Causes and Consequences, Prevention and Management*.

[51] Nicklas TA, Morales M, Linares A (2004). Children’s meal patterns have changed over a 21-year period: the Bogalusa Heart Study. *Journal of the American Dietetic Association*.

[52] Fogelholm M (2008). How physical activity can work?. *International Journal of Pediatric Obesity*.

[53] Livingstone MBE, Black AE (2003). Markers of the validity of reported energy intake. *Journal of Nutrition*.

[54] Goldberg GR, Black AE, Jebb SA (1991). Critical evaluation of energy intake data using fundamental principles of energy physiology: 1. Derivation of cut-off limits to identify under-recording. *European Journal of Clinical Nutrition*.

[57] Barba G, Troiano E, Russo P, Venezia A, Siani A (2005). Inverse association between body mass and frequency of milk consumption in children. *British Journal of Nutrition*.

[58] Zemel MB (2003). Mechanisms of diary modulation of adiposity. *Nutrition Journal*.

[59] Gibson S (2008). Sugar-sweetened soft drinks and obesity: a systematic review of the evidence from observational studies and interventions. *Nutrition Research Reviews*.

